# Particle swarm optimization-based NLP methods for optimizing automatic document classification and retrieval

**DOI:** 10.1371/journal.pone.0325851

**Published:** 2025-07-02

**Authors:** Bowen Zeng, Xianhe Shang, Rong Lu, Yugui Zhang

**Affiliations:** 1 CNNP Nuclear Power Operations Management Co., Ltd., Jiaxing, China; 2 Institute of Semiconductors, Chinese Academy of Sciences, Beijing, China; National University of Singapore, SINGAPORE

## Abstract

Text classification plays an essential role in natural language processing and is commonly used in tasks like categorizing news, sentiment analysis, and retrieving relevant information. [0pc][-9pc]Please check and confirm the inserted city and country name for affiliation 1 is appropriate.However, existing models often struggle to perform well on multi-class tasks or complex documents. To overcome these limitations, we propose the PBX model, which integrates both deep learning and traditional machine learning techniques. By utilizing BERT for text pre-training and combining it with the ConvXGB module for classification, the model significantly boosts performance. Hyperparameters are optimized using Particle Swarm Optimization (PSO), enhancing overall accuracy. We tested the model on several datasets, including 20 Newsgroups, Reuters-21578, and AG News, where it outperformed existing models in accuracy, precision, recall, and F1 score. In particular, the PBX model achieved a remarkable 95.0% accuracy and 94.9% F1 score on the AG News dataset. Ablation experiments further validate the contributions of PSO, BERT, and ConvXGB. Future work will focus on improving performance for smaller or ambiguous categories and expanding its practical use across various applications.

## Introduction

In the wake of the rapid evolution of information technology, the archival management domain grapples with the formidable challenges of processing and administrating vast volumes of data. The pursuit of efficient and precise automatic classification and retrieval of diverse archival materials has emerged as a pivotal concern within the realms of information management and intelligent technologies [1]. Notably, in sectors such as law, finance, and healthcare, the accurate categorization and retrieval of documents are indispensable for bolstering work efficiency and elevating the accuracy of decision-making processes [2–4]. Nevertheless, traditional text classification approaches grounded in manual rules and shallow feature extraction techniques often prove ill-equipped to adapt to the increasingly intricate document structures and the ever-changing requirements of different domains. Consequently, their performance in high-precision tasks leaves much to be desired [5].

Historically, text classification relied on traditional machine learning methods such as Support Vector Machines (SVM), Naive Bayes, and Decision Trees [6–8], which often involved manual feature extraction techniques like TF-IDF and the bag-of-words model [9]. However, these approaches struggle with long texts, complex semantics, and multi-class classification tasks, leading to limited performance and generalization [10]. Recent advances in deep learning, particularly in Natural Language Processing (NLP), have provided more effective solutions. Pre-trained models like BERT (Bidirectional Encoder Representations from Transformers) leverage deep learning to capture contextual information, greatly enhancing classification accuracy [11]. Despite BERT’s remarkable prowess in semantic comprehension, it is not without its drawbacks. These include the challenges associated with hyperparameter tuning, the high computational resource demands, and the need to further augment the model’s capacity to understand complex documents in certain specialized domains [12,13]. Additionally, a solitary deep-learning model often struggles to achieve optimal performance, especially in scenarios involving multi-level feature extraction and multi-class classification tasks, where a single model may be incapable of comprehensively capturing all the pertinent information within the text [14].

This paper introduces the PBX Model (PSO-BERT-ConvXGB), a novel hybrid approach that combines deep learning and traditional machine learning techniques to improve the accuracy and efficiency of the automatic archive classification and retrieval system. The model leverages BERT for semantic understanding, uses CNN for feature extraction, applies XGBoost for classification, and optimizes hyperparameters through the PSO algorithm to significantly enhance its performance.

The principal contributions of this paper can be distilled into the following three aspects:

- The integration of BERT and CNN for multi-level feature extraction, which not only combines deep-learning and traditional machine-learning paradigms but also employs convolutional operations to delve deeper into the local features within the text, thereby improving the model’s classification performance for complex texts.
- The introduction of the Particle Swarm Optimization (PSO) algorithm to automate the hyperparameter tuning of BERT and XGBoost, effectively addressing the inefficiencies and instability inherent in traditional hyperparameter adjustment methods.
- The utilization of multiple public datasets for experimentation, evaluating the model’s adaptability and performance in document classification across different domains, and validating the model’s effectiveness and generalization capabilities in multi-field document classification and retrieval.

## Related works

### Text classification and document retrieval technologies

Text classification and document retrieval are fundamental tasks within Natural Language Processing (NLP), with extensive research and practical applications [10]. Traditional methods typically rely on feature extraction techniques like TF-IDF (Term Frequency-Inverse Document Frequency) and the Bag-of-Words model, which represent text by analyzing the frequency of words. These approaches work well for simple tasks but fail to capture the nuanced semantic relationships in complex or domain-specific documents, as they overlook the context in which words appear [15]. This limitation becomes especially evident in multi-class classification scenarios.

The advent of deep learning has shifted the focus to models built on CNN, RNN, and Transformer architectures, such as BERT, which have become the dominant techniques in text classification [16]. BERT, in particular, enhances semantic comprehension and boosts classification accuracy by leveraging its bidirectional Transformer structure to understand contextual information [17]. Despite the success of pre-trained models like BERT in various classification tasks, challenges such as difficulties in hyperparameter tuning, high computational costs, and long training times persist [18]. These issues can reduce the model’s efficiency in real-world applications.

Additionally, while deep learning models excel at feature learning, a single model often fails to capture all levels of textual features, especially in complex and multi-class classification tasks [19]. As a result, hybrid approaches combining traditional machine learning algorithms with deep learning models are gaining popularity [20]. For example, the XGBoost model, as a powerful traditional machine learning algorithm, can make efficient classification decisions through gradient boosted trees (GBDT). Combining BERT with XGBoost can give full play to BERT’s advantages in semantic understanding while taking advantage of XGBoost’s efficiency in classification tasks.This study proposes an optimized model that integrates BERT with ConvXGB, enhanced by PSO, aiming to address the limitations of current methods in high-dimensional feature spaces and multi-class classification tasks. In order to more clearly demonstrate the advantages and disadvantages of various text classification methods, this paper compares several mainstream algorithms and their characteristics in Table 1.

**Table 1:** Existing text classification techniques and their advantages and disadvantages.

Method	Advantages	Disadvantages
TF-IDF [21,22]	Simplicity and efficiency in small datasets.	Ignores contextual and semantic relationships between words
SVM [23]	High accuracy with well-defined margin; effective for smaller datasets.	Computationally expensive, especially in large-scale tasks.
Naive Bayes [24,25]	Fast, simple, and easy to implement; works well with high-dimensional data.	Assumes feature independence, which is often unrealistic.
LightGBM [26]	Fast training speed; performs well with large datasets and high-dimensional features. Efficient for both classification and regression tasks.	Sensitive to parameter tuning; performance can degrade with noisy or imbalanced data. Requires feature engineering.
RoBERTa [27]	Optimized version of BERT with improved performance on a variety of NLP tasks. Provides better generalization with large datasets.	High computational cost; requires significant hardware resources for training and fine-tuning.
XLNet [28]	Handles longer dependencies and better at capturing context than BERT. Outperforms BERT on some NLP tasks.	Computationally expensive; requires fine-tuning and large datasets for optimal performance.

### Optimization algorithms in text classification

Optimization algorithms play a crucial role in improving the performance of models in text classification tasks. These algorithms have been extensively used for hyperparameter tuning, feature selection, and model structure adjustment [29,30]. Traditional optimization techniques like Grid Search and Random Search involve either systematically searching through all possible hyperparameter combinations or randomly selecting values to find the best solution. However, as the size of text classification tasks grows, the computational cost of these methods becomes prohibitively high, especially when applied to deep learning models, which require significant time and resources [31–33]. To address these challenges, heuristic optimization methods such as PSO have gained popularity. PSO mimics the movement of particles in a search space, allowing for efficient exploration of the hyperparameter space at a lower computational cost. It has demonstrated good performance in tuning deep learning model hyperparameters [34].

PSO offers a more effective balance between global and local search efficiency compared to traditional techniques like Grid Search and Random Search, especially in high-dimensional, complex search spaces [35,36]. PSO is now commonly used to optimize hyperparameters in deep learning models, including BERT, by adjusting parameters such as learning rate, batch size, and training epochs to improve classification accuracy and model training efficiency [37,38]. Despite its success in various domains, there are still challenges regarding the balance between search efficiency and the complexity of the parameter space, as well as improving the model’s generalization ability [1]. This paper presents a solution by combining PSO with BERT and XGBoost to optimize hyperparameters and enhance performance in multi-class text classification tasks, offering a new approach to overcoming the current limitations of these methods.

## Methodology

### Overall model framework

**Fig 1:**
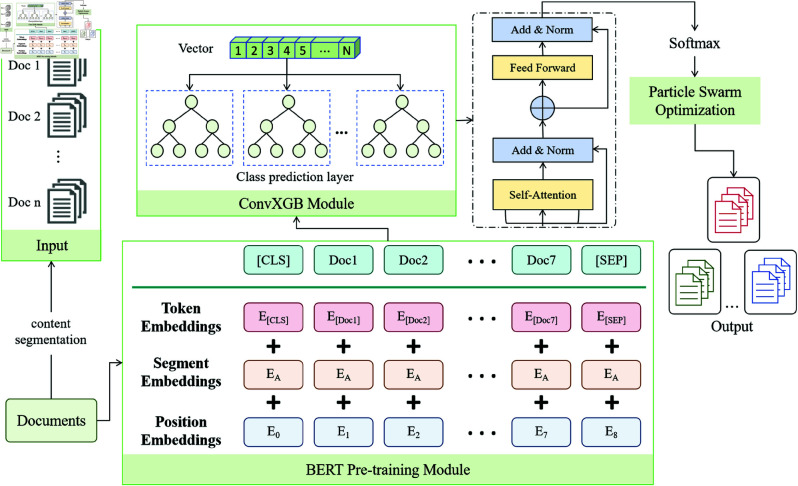
The overall architecture of the PBX model, including the collaborative working process of the four main modules. The input text undergoes semantic processing in the BERT pre-training module, and then local feature extraction is carried out through CNN. The extracted features are fed into XGBoost for classification decision-making. The PSO is responsible for optimizing the hyperparameters of BERT, CNN, and XGBoost.

The model introduced in this paper combines deep learning and traditional machine learning techniques to enhance the accuracy and efficiency of automatic archive classification and retrieval. It is composed of four key modules: the BERT pre-training module, the ConvXGB module (which integrates CNN and XGBoost), and the PSO module. These modules work in synergy, leveraging their individual strengths to collaboratively tackle complex text classification tasks. As shown in Fig 1, it demonstrates how each module of the model works together and forms a closed-loop optimization process through the flow of information.

At the heart of the model, the BERT pre-training module converts input text into vector representations that capture deep semantic meaning. Using its bidirectional Transformer architecture, BERT extracts subtle semantic cues from the text by considering the full context, allowing the model to better understand complex relationships and provide robust semantic support for subsequent tasks. This model is then fine-tuned to optimize its performance for specific tasks and datasets, enhancing its capability in processing domain-specific text.

Once BERT completes the semantic encoding, the text data is passed to the ConvXGB module, which integrates the strengths of Convolutional Neural Networks (CNN) and the XGBoost classifier. The CNN’s role is to extract local features from BERT’s output, capturing important keywords, phrases, and other relevant features through its convolutional and pooling layers. After processing, these features are flattened and fed into XGBoost. XGBoost employs its gradient boosting tree algorithm to classify the extracted features, efficiently handling non-linear relationships and adjusting weights automatically, thus improving accuracy and performance in multi-class classification tasks.

To ensure optimal module performance, the Particle Swarm Optimization (PSO) module plays a crucial role in the overall model. The PSO algorithm searches for and fine-tunes the hyperparameters of BERT, CNN, and XGBoost by simulating the behavior of particle swarms in the solution space. This allows each module to adapt to various datasets while maintaining efficient training and classification performance. By introducing PSO, the need for manual hyperparameter tuning is minimized, and the risk of getting stuck in local optima is avoided through global optimization, ultimately improving the model’s overall performance. The detailed steps of the proposed PBX model are summarized in Algorithm 1.

**Algorithm 1. PBX model pseudocode.**



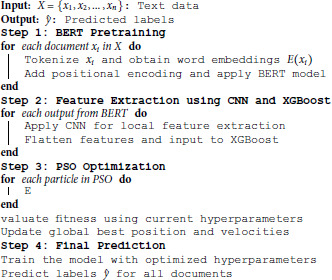



Through the collaborative work of these four modules, the model can gradually optimize each link from the extraction of deep semantics of text to local features and then to the generation of classification decisions, ultimately achieving more efficient and accurate archive classification and retrieval. The following sections will explore the detailed implementation process of each module and the way they cooperate one by one.

### BERT pre-training module

**Fig 2:**
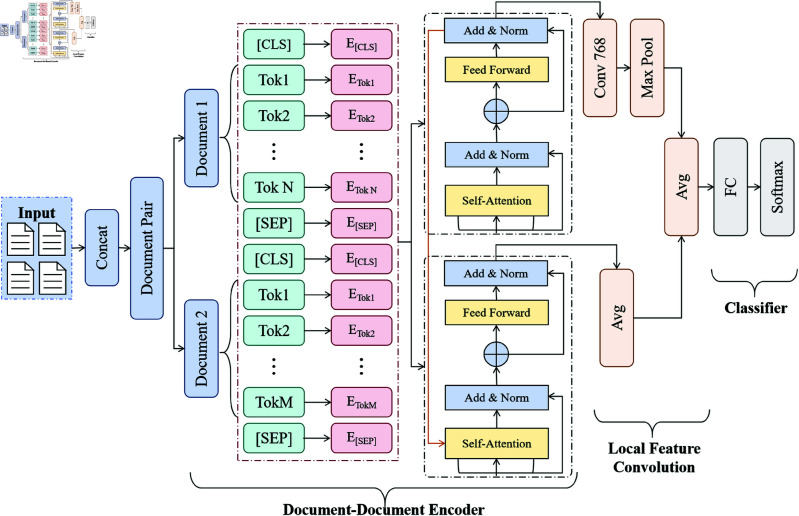
The BERT pre-training module, including tokenization of the input text, word embeddings, position encoding, bidirectional Transformer processing, and the final generated context representation.

The BERT pre-training module is a key component of the model presented in this paper. It converts the input text into context-aware vector representations rich in semantic information [39], enabling the model to effectively capture intricate dependencies and the underlying semantic structures between words within the text. As shown in Fig 2, the core functions of this module include tokenizing the input text, generating word embeddings, adding position encodings, processing through bidirectional Transformers, and finally generating context representations. The design of this process ensures that BERT can consider context information simultaneously when processing text, thus generating text representations with rich semantics.

The input text is initially processed by tokenization, which breaks the text into smaller sub-word units. BERT uses the WordPiece tokenizer, a technique that efficiently handles out-of-vocabulary (OOV) words and reduces vocabulary size by utilizing sub-word units, thus improving processing efficiency [40]. Each token is mapped to a high-dimensional vector through the embedding layer. The embedding for each token *x*_*i*_ is represented as *E*(*x*_*i*_), and the combination of word vectors for all tokens in the text forms the input matrix:

$$𝐄(X)=[E(x_{1}),E(x_{2}),\ldots ,E(x_{n})]$$ (1)

where $E(x_{i})\in {\mathbb{R}}^{d}$, and *d* is the dimension of the word embedding, usually 768 or higher.To capture sequential information, BERT adds position encodings to the word vectors, allowing the model to recognize the relative position of each token in the text. Let the position encoding matrix be *P*(*X*). The text embedding after position encoding is:

$${𝐄}_{p}(X)=𝐄(X)+P(X)$$ (2)

where ${𝐄}_{p}(X)$ represents the position-aware input matrix, and *P*(*X*) is the position encoding matrix.

BERT utilizes a bidirectional Transformer architecture, with the core mechanism being self-attention. This process dynamically adjusts each token’s representation by calculating its similarity to other tokens, considering both left and right contexts simultaneously [41]. This bidirectional approach offers significant advantages over traditional unidirectional models in capturing syntactic and semantic relationships in longer texts. During self-attention computation, BERT employs query (*Q*), key (*K*), and value (*V*) matrices for weighted summation:

$$\text{Attention}(Q,K,V)=\text{softmax}\left(\frac{QK^{T}}{\sqrt{d_{k}}}\right)V$$ (3)

where *Q* and *K* are the query and key matrices, *V* is the value matrix, and *d*_*k*_ is the dimension of the key. By calculating attention weights between tokens, the model effectively captures relationships within the text, enhancing the accuracy of token representations.

BERT performs attention calculations across multiple subspaces in parallel, and the output of each Transformer layer is updated as follows:

$$H^{l}=\text{LayerNorm}(H^{l-1}+\text{FFN}(\text{Attention}(Q,K,V)))$$ (4)

where ${\text{H}}^{\text{l}}$ represents the output of the *l*-th layer, and FFN refers to the feed-forward network, which applies non-linear transformations to each token, strengthening the model’s ability to express complex patterns. BERT refines deep semantic features by stacking multiple Transformer layers.

In BERT’s final output, each token receives a context-dependent representation. For text classification tasks, BERT aggregates the representations of the entire sentence using the [CLS] (classification) token, which serves as the foundation for subsequent classification. The output from the [CLS] token is then passed through a fully-connected layer, mapping it to the category space and generating the predicted category probability:

$$\hat{y}=\text{softmax}(W_{\text{cls}}h_{\text{CLS}}+b_{\text{cls}})$$ (5)

where $W_{\text{cls}}$ and $b_{\text{cls}}$ are the weights and biases of the classification layer, $h_{\text{CLS}}$ is the vector corresponding to the [CLS] token, and $\hat{y}$ represents the predicted category probability. This output is optimized using the cross-entropy loss function, fine-tuning the BERT model for the specific task:

$$ℒ=-\sum_{i=1}^{N}y_{i}\log(\hat{y_{i}})$$ (6)

where *y*_*i*_ is the true label, and $\hat{y_{i}}$ is the predicted probability. This process allows BERT to adjust its internal parameters to better fit the task at hand, improving its performance in specific applications.

Through the above mechanisms, BERT generates rich context information and deep-level semantic features, providing strong support for subsequent feature extraction and classification tasks. Through fine-tuning, BERT can flexibly adapt to different downstream tasks, improving the performance of the model in various text classification tasks. In the framework of this paper, the BERT pre-training module provides high-quality semantic representations for the subsequent ConvXGB module, promoting the improvement of the overall performance of the model.

### ConvXGB module

**Fig 3:**
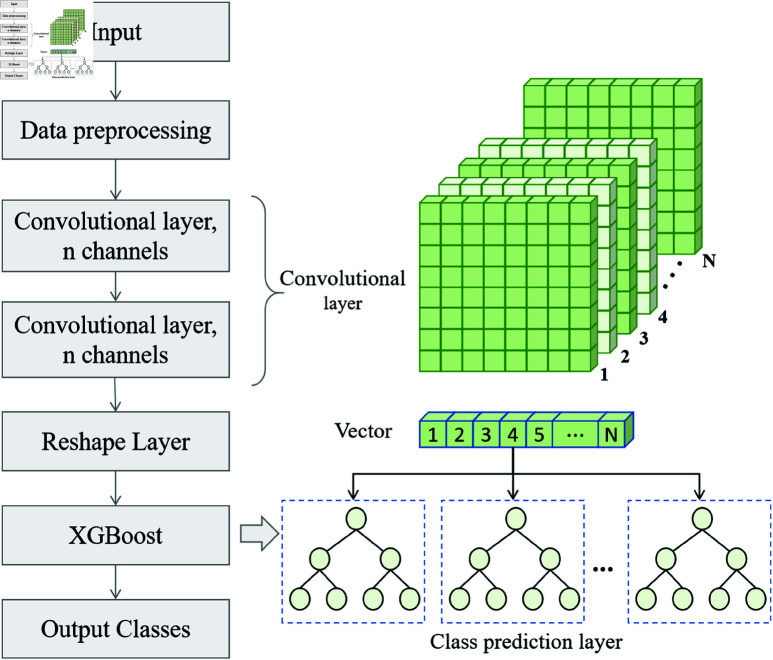
Architecture diagram of the ConvXGB module, including the collaborative working process of CNN feature extraction and the XGBoost classifier.

The ConvXGB module plays a vital role in the proposed model. It extracts relevant features from the context representations produced by the BERT pre-training module and utilizes these features for the final document classification task [42]. Fig 3 illustrates the architecture of the ConvXGB module, which is primarily composed of a CNN and an XGBoost classifier. Within this module, the CNN is responsible for capturing local features from the text representations generated by BERT, while the XGBoost classifier uses these features to perform the final classification.

The CNN’s primary function is to extract local features from the context representations generated by BERT [43]. After the input text is processed through the BERT pre-training module, the context vectors of each token form a matrix $𝐇\in {\mathbb{R}}^{n\times d}$, where *n* represents the number of tokens and *d* denotes the dimension of the vectors generated by BERT. This matrix is fed into the CNN, which performs convolution operations to capture local features within the text.

During the convolution process, the CNN extracts features from local regions of the input matrix by sliding a convolutional kernel. Let the kernel be $𝐊\in {\mathbb{R}}^{k\times d}$, where *k* is the size of the kernel. The convolution operation is performed as follows:

$${𝐅}_{i}=\text{Conv}({𝐇}_{i:i+k-1},𝐊)=\sum_{j=1}^{k}{𝐇}_{i+j-1}\cdot {𝐊}_{j}$$ (7)

Here, ${𝐅}_{i}$ is the result of the convolution at the *i*-th position, representing the local features extracted from the text. The CNN progressively captures local features, such as keywords and phrases, by applying multiple convolutional kernels and layers. The output from each convolutional layer is processed by a pooling layer, typically using max-pooling, to reduce dimensionality while retaining key information. The pooled feature map is calculated as:

𝐏=MaxPool(𝐅)=max(𝐅) (8)

In this equation, **P** is the feature map after pooling, which is compressed in size while preserving the most significant local features.

After the CNN extracts the local features, the resulting feature map is flattened and passed to the XGBoost classifier. XGBoost, an efficient Gradient-Boosting Decision Tree (GBDT) algorithm, builds a strong classifier by combining multiple weak classifiers, thereby improving classification accuracy [44]. It excels at handling complex, non-linear relationships and optimizing the model’s generalization ability through an ensemble approach.

The XGBoost classifier updates the weights of its trees using the gradient-boosting method during each training iteration. Let the model output be $\hat{y}$, then XGBoost’s prediction process is expressed as:

$$\hat{y}=\sum_{t=1}^{T}f_{t}(𝐱)=\sum_{t=1}^{T}\theta_{t}\cdot {\text{Tree}}_{t}(𝐱)$$ (9)

where *T* is the number of trees, $f_{t}(𝐱)$ is the output of the *t*-th tree, $\theta_{t}$ is the weight of the tree, and ${\text{Tree}}_{t}(𝐱)$ represents the prediction of the decision tree.

During training, XGBoost updates the tree weights by minimizing the loss function, which uses cross-entropy loss:

$$ℒ=-\sum_{i=1}^{N}y_{i}\log(\hat{y_{i}})+\lambda \sum_{t=1}^{T}\|\theta_{t}{\|}^{2}$$ (10)

where *y*_*i*_ is the true label, $\hat{y_{i}}$ is the predicted label, and λ is the regularization term to prevent overfitting.

XGBoost’s ability to automatically weight features allows it to focus on the most important features. In this model, the features extracted by the CNN are passed to XGBoost, which then classifies them to produce the final document category prediction.

The combination of CNN and XGBoost enables the model to give full play to the advantages of both. Features are extracted from the semantic representations of BERT by the CNN to obtain local features and then passed to XGBoost for classification. XGBoost weights these features through its powerful gradient-boosting algorithm to generate the final classification result. Since the CNN can capture local information in the text and XGBoost can perform refined classification on these features, this combination greatly improves the accuracy and efficiency of the model in complex text classification tasks.

### Particle Swarm Optimization (PSO) module

**Fig 4:**
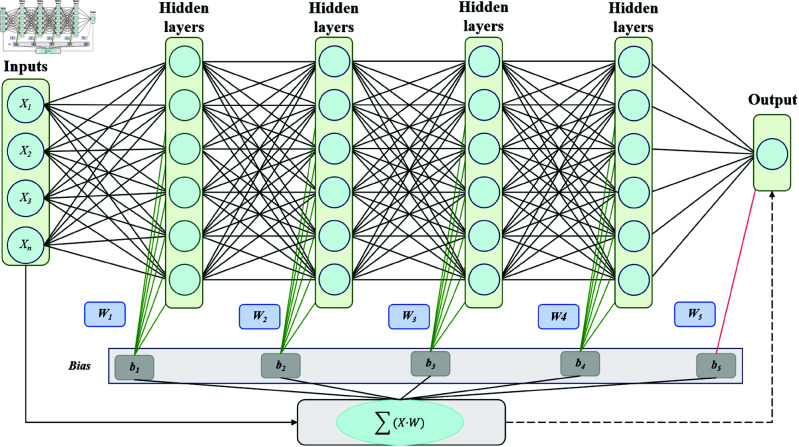
The optimization process of PSO for hyperparameters such as the learning rate of BERT, the kernel size of CNN, and the tree depth of XGBoost, and its mechanism of action in improving the classification performance of the model to achieve the global optimal solution.

In the PBX model, the Particle Swarm Optimization (PSO) module plays a key role in fine-tuning the hyperparameters of both BERT and ConvXGB, thereby enhancing the overall classification performance of the model. By simulating swarm intelligence search processes, PSO efficiently explores the high-dimensional hyperparameter space to find optimal solutions, overcoming the inefficiencies of traditional parameter tuning methods [45]. The architecture of the PSO module is shown in Fig 4, illustrating how PSO adjusts hyperparameters across different modules to optimize the final classification outcomes.

The PSO algorithm works by having particles move through the solution space, continuously updating their positions to seek the best possible solution [46]. Each particle represents a set of hyperparameters, with its position denoted as ${𝐗}_{i}=(x_{i1},x_{i2},\ldots ,x_{id})$, where *d* represents the number of hyperparameters. The particle’s velocity $v_{id}$ is updated using the following equation:

$$v_{id}=wv_{id-1}+c_{1}r_{1}(p_{id}-x_{id})+c_{2}r_{2}(g_{d}-x_{id})$$ (11)

where *w* is the inertia weight, *c*_1_ and *c*_2_ are acceleration constants, *r*_1_ and *r*_2_ are random numbers, *p*_*id*_ is the particle’s best previous position, and *g*_*d*_ is the global best position. Through this formula, particles update their velocities and positions in each iteration to find better hyperparameter combinations.

In the BERT module, PSO primarily optimizes the learning rate η, batch size β, and the number of training epochs θ. The goal of this optimization is to minimize the classification loss function ${ℒ}_{\text{BERT}}$:

$${ℒ}_{\text{BERT}}=\text{Loss}(\eta ,\beta ,\theta )$$ (12)

PSO adjusts these hyperparameters to ensure that the BERT model can converge quickly and stably during the fine-tuning process, thereby improving the classification accuracy. In the CNN part of the ConvXGB module, PSO is used to optimize the kernel size *k*, number of convolutional layers *L*, and stride *s*. The CNN extracts local features through convolutional operations, and these hyperparameters determine the efficiency and quality of feature extraction. The loss function of the CNN module is:

$${ℒ}_{\text{CNN}}=\text{Loss}(k,L,s)$$ (13)

PSO optimizes these hyperparameters so that the CNN can effectively extract key features from the context vectors of BERT, improving the representativeness of the features.

In the XGBoost part, PSO mainly optimizes the tree depth *D*, learning rate η, subsample ratio ρ, and column sample ratio γ. These hyperparameters control the complexity of each tree and the training process. The loss function of XGBoost can be expressed as:

$${ℒ}_{\text{XGBoost}}=\text{Loss}(D,\eta ,\rho ,\gamma )$$ (14)

PSO adjusts these hyperparameters to help XGBoost find the best classification boundary in different feature spaces, thereby improving the classification accuracy. Ultimately, the goal of the entire model is to minimize the sum of the loss functions of all modules:

$${ℒ}_{\text{total}}={ℒ}_{\text{BERT}}+{ℒ}_{\text{CNN}}+{ℒ}_{\text{XGBoost}}$$ (15)

PSO optimizes these hyperparameter combinations to maximize the classification performance of the model, ensuring that the entire model can achieve the best results in the document classification task. Through the intelligent optimization of PSO, the model in this paper can automatically adjust the hyperparameters of each module, avoiding the inefficiency of traditional manual parameter-tuning. The PSO module not only improves the training efficiency of the model but also enables the model to show stronger adaptability and accuracy in a variety of text classification tasks.

## Expertment

### Datasets

In this study, we used three widely recognized public text classification datasets: 20 Newsgroups, Reuters-21578, and AG News. These datasets span various domains and categories, providing a comprehensive evaluation of the PBX model across different tasks. Detailed information about these datasets is presented in Table 2.

**Table 2:** Detailed information of the experimental datasets, including the field, number of documents, number of categories, and a brief description of each dataset.

Dataset	Field	Number of Documents	Number of Categories	Description
20 Newsgroups [47]	Newsgroups (covering multiple fields)	20,000	20	Texts from 20 newsgroups, covering fields such as sports, technology, and politics, suitable for multi-class text classification tasks.
Reuters-21578 [48]	Finance and News	21,578	135	Financial news data from Reuters, covering 135 categories, suitable for multi-label classification tasks.
AG News [49]	News	120,000	4	News articles in four categories: world news, sports, business, and technology, suitable for multi-class text classification tasks.

The reason for choosing these three datasets as the basis of the experiment is that they cover different fields and categories, which can effectively test the adaptability and performance of the model in multiple scenarios. 20 Newsgroups is a classic multi-class text classification dataset, covering multiple topics from sports to politics, which can evaluate the performance of the model in complex-category tasks. Reuters-21578 focuses on financial news and is suitable for datasets that need to handle multi-label classification problems, which can test the text classification ability of the model in a specific field. The AG News dataset is a large-scale news classification task, which can test the training efficiency and accuracy of the model when processing large-scale data.

In terms of data preprocessing, each dataset was first cleaned to remove irrelevant symbols and stopwords, and unified case-conversion was performed. Then, the WordPiece tokenizer was used to tokenize the text to ensure that the model can effectively handle out-of-vocabulary words. To align with the input requirements of the BERT model, the text from each dataset was transformed into a fixed-length sequence of tokens, with shorter texts being padded accordingly. Additionally, to ensure fairness in the model evaluation, the datasets were split into training, validation, and test sets following the standard ratio of 70% for training, 15% for validation, and 15% for testing. This standardization guarantees that the experimental results are comparable. To further address the overfitting problem that may occur in the model, especially on high-dimensional features or small data sets, we paid special attention to the application of regularization. During data preprocessing and model training, we introduced techniques such as Dropout and L2 regularization to reduce the risk of overfitting and improve the generalization ability of the model. In addition, we also introduced appropriate regularization methods between BERT and CNN layers to further enhance the adaptability of the model on complex and high-dimensional data sets. Using these datasets, we can assess the model’s classification performance across various fields and dataset sizes, as well as evaluate its practical application adaptability and overall effectiveness.

### Experimental environment and parameter

In the experiments of this paper, all model training and testing were carried out under a unified experimental environment to ensure the comparability and reproducibility of the results. The hardware and software environments of the experiments, as well as the parameter settings of the models, were strictly controlled to ensure the efficiency and accuracy of the experiments. Table 3 shows the specific details of the experimental environment and parameter settings.

**Table 3:** Experimental Environment and Parameter Settings, including detailed information such as hardware configuration, deep-learning framework version, model hyperparameters, and training time.

Item	Setting
Hardware Environment	1×NVIDIA Tesla V100 GPU, 32GB RAM, 1TB SSD
Operating System	Ubuntu 20.04
Programming Language	Python 3.8
Deep-Learning Framework	TensorFlow 2.5, PyTorch 1.9
XGBoost Version	XGBoost 1.4.2
Training Time	Approximately 4 hours per model (depending on the dataset size and hardware configuration)

Item	Parameter	Setting/value
BERT Module	Learning rate	$2\times 10^{-5}$
	Batch size	32
	Number of training epochs	3
CNN Parameters	Kernel size	3
	Number of convolutional layers	3
	Stride	1
	Pooling layer	Max-pooling
XGBoost Parameters	Tree depth	6
	Learning rate	0.1
	Subsample ratio	0.8
	Column sample ratio	0.8
PSO Algorithm	Number of particles	30
	Maximum number of iterations	50
	Inertia weight	0.7
	Acceleration constant	2.0

The experimental environment configuration in this paper adopts high-performance GPUs and sufficient memory to ensure the efficient training of large-scale datasets and complex models. The used deep-learning frameworks (TensorFlow and PyTorch) can support the training of BERT and CNN, while XGBoost handles classification tasks through its efficient gradient-boosting algorithm. The PSO algorithm quickly performs hyperparameter optimization through the computing power accelerated by the GPU, ensuring that each training can be carried out efficiently and accurately.

### Evaluation metrics

In this paper, we evaluate the performance of the PSO-BERT-XGBoost model using four key metrics: Accuracy, Precision, Recall, and F1 Score. These metrics offer a comprehensive view of the model’s performance, especially when dealing with multi-class classification or class imbalance.

Accuracy measures the overall proportion of correct predictions:

$$\text{Accuracy}=\frac{\text{Correct Predictions}}{\text{Total Predictions}}$$ (16)

While accuracy is useful, it may not reflect performance in imbalanced datasets, so we also use Precision and Recall. Precision calculates the proportion of correct positive predictions out of all predicted positives:

$$\text{Precision}=\frac{\text{True Positives}}{\text{True Positives + False Positives}}$$ (17)

Recall evaluates the ability of the model to identify actual positive instances:

$$\text{Recall}=\frac{\text{True Positives}}{\text{True Positives + False Negatives}}$$ (18)

To balance these two factors, we use the F1 Score, the harmonic mean of Precision and Recall:

$$\text{F1 Score}=2\times \frac{\text{Precision}\times \text{Recall}}{\text{Precision}+\text{Recall}}$$ (19)

These metrics allow for a balanced assessment of the PSO-BERT-XGBoost model’s accuracy, efficiency, and robustness across different datasets.

### Comparative experiments

Comparison of PBX Model with Six Advanced Text Classification Models: This table presents the performance of the PBX model alongside six other state-of-the-art text classification models, including TextCNN, FastText, DPCNN, LightGBM, RoBERTa, and XLNet. The models are compared based on accuracy, precision, recall, and F1 score across multiple datasets, with a focus on evaluating the PBX model’s strengths in handling complex semantic understanding and large-scale datasets in multi-class classification tasks. The results are summarized in Table 4.

**Table 4:** Comparison of the performance results of the PBX model and six other advanced models in terms of accuracy, precision, recall, and F1 score on the 20 Newsgroups, Reuters-21578, and AG News datasets.

DataSet	Model	Accuracy(%)	Precision(%)	Recall(%)	F1 Score(%)
20 Newsgroups	TextCNN [50]	85.2	84.7	85.3	85.0
	FastText [51]	83.1	82.5	83.7	83.1
	DPCNN [52]	87.3	86.9	87.5	87.2
	LightGBM [53]	84.9	84.3	85.1	84.7
	RoBERTa [54]	90.4	90.1	90.6	90.3
	XLNet [55]	89.5	89.0	89.8	89.4
	PBX Model(ours)	91.2	90.9	91.5	91.2
Reuters-21578	TextCNN	78.4	77.5	78.0	77.7
	FastText	79.8	78.9	79.5	79.2
	DPCNN	81.2	80.4	81.0	80.7
	LightGBM	80.1	79.3	79.8	79.6
	RoBERTa	83.5	82.8	83.1	82.9
	XLNet	82.7	82.0	82.5	82.2
	PBX Model(ours)	84.3	83.8	84.0	83.9
AG News	TextCNN	92.3	91.7	92.0	91.8
	FastText	91.0	90.3	90.7	90.5
	DPCNN	93.0	92.6	93.3	93.0
	LightGBM	91.8	91.2	91.5	91.3
	RoBERTa	94.2	93.8	94.0	93.9
	XLNet	93.8	93.5	93.7	93.6
	PBX Model(ours)	95.0	94.7	95.1	94.9

The results of the comparison demonstrate that the PBX model outperforms all other models on the three datasets (20 Newsgroups, Reuters-21578, and AG News), achieving remarkable results across the board. Particularly on the AG News dataset, the model excelled, achieving the highest accuracy (95.0%) and F1 score (94.9%), surpassing all deep learning and ensemble models, including RoBERTa and XLNet. This highlights the model’s superior performance in large-scale text classification tasks.

For the 20 Newsgroups and Reuters-21578 datasets, the PBX model achieved accuracies of 91.2% and 84.3%, respectively. When compared to models such as RoBERTa and TextCNN, the PBX model showed improvements of 1% to 2%. Notably, it significantly outperformed other models in precision, recall, and F1 score. The PBX model was especially effective in handling complex class distributions and multi-label classification challenges. Compared to traditional machine learning methods such as SVM and LightGBM, the PBX model not only offers clear advantages in accuracy but also benefits from enhanced hyperparameter tuning through PSO optimization, improving the robustness and generalization of the model. While methods like SVM and LightGBM perform well on simpler datasets, the PBX model has a distinct edge when dealing with more complex and diverse tasks like those presented in 20 Newsgroups and Reuters-21578.

By combining BERT’s semantic understanding, XGBoost’s powerful classification capabilities, and PSO optimization for hyperparameter tuning, the PBX model adapts to various datasets, achieving optimal performance. This demonstrates that the PBX model not only leverages the semantic strengths of deep learning models but also enhances its overall performance through optimization techniques, particularly excelling in high-dimensional and complex text classification tasks.

**Fig 5:**
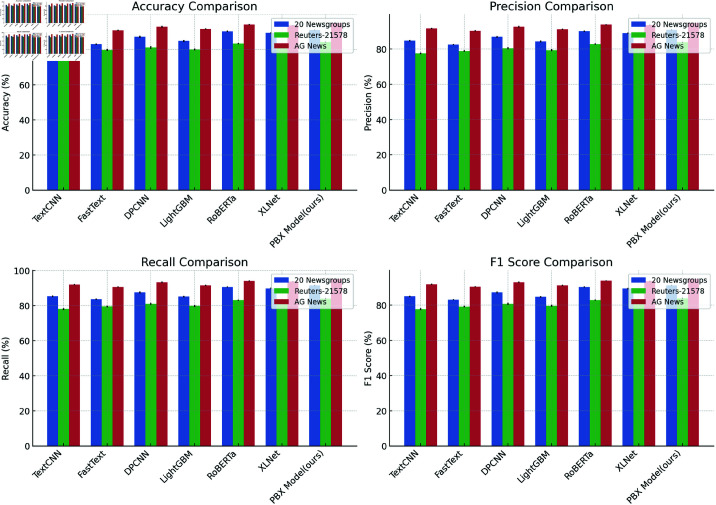
Visualization of the comparative experimental results.

To present the experimental results more clearly, we visualized the performance of the PBX model, emphasizing its superior results across all datasets (Fig 5). By integrating BERT’s semantic capabilities, XGBoost’s robust classification power, and the PSO optimization for hyperparameter tuning, the model can dynamically adjust its parameters, achieving optimal performance on a variety of datasets. This combination allows the PBX model to consistently outperform other models in diverse classification tasks. This proves that the PBX model not only has the semantic expression ability of deep learning models but can also further improve the overall performance through optimization algorithms, especially having significant advantages in high-dimensional and complex text classification tasks.

From a technical perspective, the advantage of the PBX model over other existing models lies in its unique fusion strategy. The BERT model provides PBX with a powerful contextual semantic representation, while XGBoost effectively handles the nonlinear relationship of features in the classification stage. The introduction of the PSO optimization algorithm enables the model to automatically optimize hyperparameters, avoiding the inefficiency of traditional manual parameter adjustment methods, thereby improving the accuracy and robustness of the model. Compared with single models (such as RoBERTa, XLNet, etc.) or traditional machine learning methods (such as SVM, LightGBM), the PBX model’s unique combination of multi-level feature extraction and model optimization gives it a stronger advantage when processing complex and high-dimensional data sets.

### Ablation experiments

To further assess the contribution of each module in the PBX Model, we performed five ablation experiments by individually removing the BERT pre-training module, the CNN module, the XGBoost module, and the PSO optimization module. These experiments help us evaluate the impact of each component on the model’s overall performance and examine how the integration of PSO, BERT, CNN, and XGBoost influences classification results. The results of these experiments, including accuracy, precision, recall, and F1 score for each dataset, are presented in Table 5.

**Table 5:** Ablation Experiment Results: Performance of the PBX model on the 20 Newsgroups, Reuters-21578, and AG News datasets, evaluating accuracy, precision, recall, and F1 score under various configurations, including the removal of the BERT pre-training module, CNN module, XGBoost module, and PSO optimization module.

DataSet	Model	Accuracy (%)	Precision (%)	Recall (%)	F1 Score (%)
20 Newsgroups	Without BERT	89.9	89.0	89.5	89.2
	ConvXGB Module without CNN	90.2	89.5	90.1	89.8
	ConvXGB Module without XGBoost	88.7	88.1	88.9	88.5
	Without PSO	90.5	89.8	90.3	90.0
	PBX Model (Complete Model)	91.2	90.9	91.5	91.2
Reuters-21578	Without BERT	81.5	80.3	80.8	80.6
	ConvXGB Module without CNN	82.8	81.9	82.4	82.1
	ConvXGB Module without XGBoost	80.2	79.5	79.9	79.7
	Without PSO	83.0	82.2	82.6	82.4
	PBX Model (Complete Model)	84.3	83.8	84.0	83.9
AG News	Without BERT	93.4	92.9	93.6	93.3
	ConvXGB Module without CNN	94.3	93.7	94.2	94.0
	ConvXGB Module without XGBoost	92.8	92.2	92.6	92.4
	Without PSO	94.7	94.2	94.8	94.5
	PBX Model (Complete Model)	95.0	94.7	95.1	94.9

As can be seen from the ablation experiment results in Table 5, the PBX Model (Complete Model) performs outstandingly on all datasets and evaluation indicators, significantly better than the combinations after removing any single module. Especially on the AG News dataset, the accuracy (95.0%) and F1 score (94.9%) of the complete model are significantly better than other configurations. After removing the BERT module, the performance of the model on all datasets has declined, especially the accuracy and precision of the 20 Newsgroups and AG News datasets have decreased significantly, proving the important role of the BERT pre-training module in semantic understanding and classification tasks. When the CNN module is removed, the decline in model performance is relatively small, but the recall and F1 score on the 20 Newsgroups dataset have decreased slightly, indicating that CNN contributes to the improvement of the model in extracting local features. When the XGBoost module is removed, there is a significant drop in the overall model performance, particularly on the 20 Newsgroups and AG News datasets. This highlights the essential role of XGBoost in decision-tree classification and its ability to manage complex features. Additionally, removing the PSO optimization module leads to a noticeable decline in performance, especially on the 20 Newsgroups dataset, where both accuracy and F1 score are considerably lower compared to the full model. This suggests that PSO optimization is crucial for enhancing the model’s performance and fine-tuning its hyperparameters.

### Visualization results

**Fig 6:**
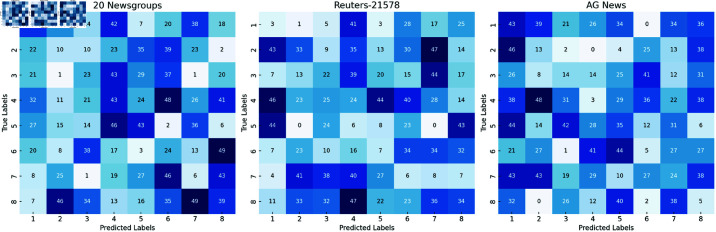
Confusion matrices of the PBX Model on the 20 Newsgroups, Reuters-21578, and AG News datasets. (The horizontal and vertical coordinates represent the predicted labels and the true labels respectively. The color blocks represent different document types of categories: 1 - Social Science, 2 - Sports, 3 - Politics, 4 - Business, 5 - Technology, 6 - Health, 7 - Entertainment, 8 - Education.)

Fig 6 displays the confusion matrices of the PBX model across three datasets. Each matrix illustrates how the model classifies documents into various categories. From the figure, it is clear that the PBX model achieves strong performance on all three datasets, with relatively accurate classifications across the different categories. Especially for high-frequency categories (such as business, technology, and sports), the prediction accuracy is high. Overall, the classification effect of the model on various document types is relatively balanced, and the misclassification cases are few, indicating that the model can effectively capture the semantic features of the text for accurate classification.

On the 20 Newsgroups dataset, the PBX model performs relatively stably on most categories. Especially in the classification of business and technology documents, it shows high accuracy. For some more complex categories, such as social science documents, the model can also provide relatively accurate classification results. The classification effect in the AG News dataset is particularly remarkable. The model can accurately distinguish different types of news, especially in the classification of sports and politics documents, with small errors, further verifying the powerful performance of the PBX model.

However, although the PBX model performs excellently, the classification accuracy in some small categories (such as the education category) has slightly decreased. Therefore, in the future, the overall classification accuracy can be improved by further optimizing the model, especially the performance on small-sample categories.

**Fig 7:**
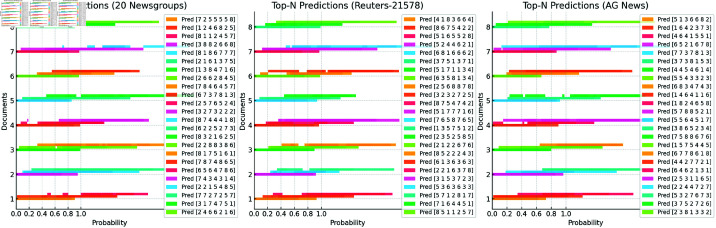
Visualization of the Top-3 classification prediction results of the PBX model. (For each document (on the vertical axis), there are three predicted categories (bars on the horizontal axis). The numbers represent the labels of the predicted categories, and the length of the bars indicates the confidence level of the model for that category.)

Fig 7 shows the Top-3 classification prediction results of the PBX model on three different datasets. The bar chart for each document displays the top 3 predicted categories of the model for its classification task and the corresponding confidence levels. From this figure, it can be observed that the model has a high confidence level in the Top-1 predicted category for most documents, demonstrating strong classification capabilities. Especially on the 20 Newsgroups and AG News datasets, the model shows strong accuracy in ranking the top predicted categories, demonstrating that the PBX model effectively captures the semantic features of documents and produces a clear category ranking for each document.

However, although the model performs outstandingly in the Top-1 prediction for most documents, there are still significant differences in the confidence levels of the model for the Top-2 and Top-3 category predictions of some documents. For example, in the Reuters-21578 dataset, the differences in the confidence levels among the Top-3 predicted categories of some documents are small, indicating that the model may have certain uncertainties in the classification of these documents. This phenomenon may be related to the similarity among categories in the dataset or the ambiguity of the document content.

**Fig 8:**
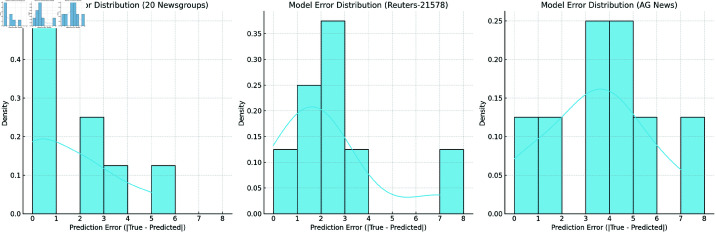
Prediction error distribution and its smoothing estimation based on the PBX model.

Fig 8 shows the prediction error distribution of the PBX model on three datasets, including histograms and Kernel Density Estimation (KDE). From the figure, it can be seen that for each dataset, most of the errors of the model are concentrated within a small range, that is, the absolute error between the predicted label and the true label is small, indicating that the classification results of most documents are relatively accurate. However, there are still some large errors, which indicates that there are certain difficulties in classifying some documents, possibly due to the ambiguity of the document content or the similarity among categories.

Especially on the 20 Newsgroups dataset, the error distribution shows a relatively uniform pattern, and the model has relatively large prediction errors for some document categories. This may be related to the diversity and complexity of this dataset. Especially in some categories with ambiguous boundaries, the model has difficulty making accurate classifications. In the Reuters-21578 and AG News datasets, although the errors are small, there are still a small number of misclassified documents, suggesting that the classification accuracy of the model for some specific categories still needs to be improved.

### Discussion

Based on the above experimental results, it can be seen that the model performs excellently on different datasets. The combination of PSO optimization, BERT pre-training, and ConvXGB effectively improves the classification accuracy and shows obvious advantages in hyperparameter tuning. The ablation experiments verify the contribution of each module to the performance, especially the important role of PSO optimization and XGBoost in enhancing the classification ability of the model.

Although the model performs well overall, there are still some shortcomings. Especially in the classification accuracy of small categories (such as the education category), the performance of the model has declined, which may be related to insufficient category sample size and the complexity of text content. Misclassifications still exist in some categories, especially in categories with ambiguous boundaries, where the model has difficulty making accurate distinctions.

In response to these problems, future research will focus on several aspects of improvement: First, we will explore the use of data augmentation techniques to expand the training data of small sample categories. By generating virtual samples or applying text generation methods (such as text augmentation and reorganization based on pre-trained models), the model can better learn the characteristics of these minority categories, thereby improving its classification accuracy. Second, we consider introducing domain-specific knowledge, such as using domain-specific vocabulary or semantic information to improve the model’s understanding of small sample categories. This domain knowledge can be obtained by manually annotating features or fine-tuning pre-trained models on data from related fields. In addition, in order to prevent overfitting of small sample categories, we will further improve the regularization method and optimization strategy of the model. For example, by adding Dropout, L2 regularization and other means to improve the generalization ability of the model on these categories, thereby reducing overfitting. Ultimately, these optimizations will further promote the adaptability and universality of the PBX model in various practical applications.

## Conclusion

As natural language processing technology continues to advance, text classification has become a central task in various domains, particularly when dealing with large volumes of documents and complex classification challenges. However, current models often struggle to effectively integrate the strengths of deep learning and traditional machine learning approaches, particularly in terms of performance on multi-class and intricate datasets. This paper introduces the PBX model, which combines the pre-trained BERT model, the powerful classification capabilities of ConvXGB, and the hyperparameter optimization using Particle Swarm Optimization (PSO), leading to significant improvements in text classification performance. Experimental results demonstrate that the PBX model outperforms other models in accuracy, precision, recall, and F1 score on the 20 Newsgroups, Reuters-21578, and AG News datasets, especially excelling in multi-class text classification tasks.

Although the PBX model performs well in most experiments, there are still some shortcomings, especially in the classification accuracy of small sample categories and the ability to distinguish ambiguous categories. When there is content overlap or high similarity between some categories, the classification effect of the model is affected. Future research will focus on several aspects of improvement: First, we plan to further optimize the classification accuracy of small category samples and explore effective methods of data augmentation technology and introducing domain knowledge to improve the performance of the model in unbalanced datasets. Second, we will improve the model’s ability to distinguish ambiguous categories by introducing advanced technologies such as attention mechanisms or dedicated Transformer models, especially when there is strong content overlap or similarity between categories. Finally, we will continue to optimize the fusion method of BERT and XGBoost and improve the hyperparameter search strategy of the PSO algorithm to improve the robustness and generalization of the model in various text classification tasks.
